# *QuickStats:* Percentage of Suicides* and Homicides^†^ Involving a Firearm Among Persons Aged ≥10 Years, by Age Group — National Vital Statistics System, United States, 2020^§^

**DOI:** 10.15585/mmwr.mm7119a5

**Published:** 2022-05-13

**Figure:**
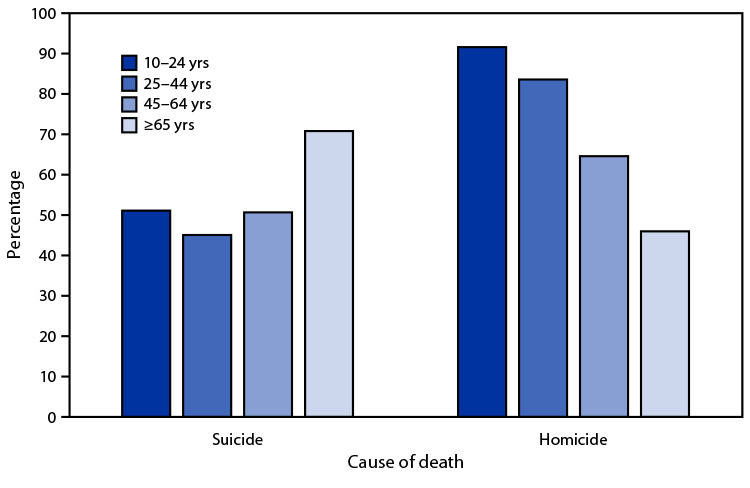
In 2020, among persons aged ≥10 years, the percentage of suicide deaths that involved a firearm was lowest among those aged 25–44 years (45.1%) and highest among those aged ≥65 years (70.8%). The percentage of homicide deaths that involved a firearm decreased with age, from 91.6% among those aged 10–24 years to 46.0% among those aged ≥65 years. Persons aged ≥65 years had the highest percentage of suicide deaths that involved a firearm but the lowest percentage of homicide deaths that involved a firearm.

**Source:** National Vital Statistics System, Mortality Data, 2020. https://www.cdc.gov/nchs/nvss/deaths.htm

For more information on this topic, CDC recommends the following links: https://www.cdc.gov/suicide and https://www.cdc.gov/violenceprevention

